# Maternal outcomes of severe preeclampsia and eclampsia and associated factors among women admitted at referral hospitals of amhara regional state, institutional-based cross-sectional study, North-West Ethiopia

**DOI:** 10.3389/fgwh.2025.1555778

**Published:** 2025-03-27

**Authors:** Misganaw Fikrie Melesse, Getie Lake Aynalem, Martha Berta Badi, Bewket Yeserah Aynalem

**Affiliations:** ^1^ Department of Midwifery, Debre Markos University, Debre Markos, Ethiopia; ^2^ School of Midwifery, University of Gondar, Gondar, Ethiopia

**Keywords:** preeclampsia, eclampsia, maternal outcomes, pregnant women's health, Ethiopia

## Abstract

**Introduction:**

Severe preeclampsia/eclampsia is a multi-systemic pregnancy condition that manifests after 20 weeks of gestation and is linked to a high global rate of maternal morbidity and mortality. It is responsible for 11%–14% of maternal mortality globally and is the second most frequent direct obstetrical cause of death. This study aimed to assess maternal outcomes of severe preeclampsia/eclampsia and associated factors in mothers admitted to referral hospitals in the Amhara Regional State of North West Ethiopia.

**Methods:**

An institutionally based cross-sectional investigation on the outcomes of severe preeclampsia/eclampsia in mothers was carried out from April 1 to September 30, 2018. Using the census sampling technique, 456 study participants were included in the study. Data were coded, verified, and imported into Epi-info version 7.2 before being exported and analyzed in SPSS version 26. To determine the determinants of maternal outcomes of severe preeclampsia or eclampsia, binary logistic regression was employed, with a significance level of 95% confidence interval of odds ratio at *p*-value 0.05 or below.

**Results:**

Overall, severe preeclampsia and eclampsia were shown to have unfavorable maternal outcomes in 37.7% (95% CI: 32.8%, 42.3%). The following variables had statistically significant associations with unfavorable maternal outcomes of severe preeclampsia and eclampsia: educational status (AOR = 4.5, 95% CI: 1.95, 12.31), residence (AOR = 2.1, 95% CI: 1.17, 3.72), monthly family income (AOR = 2.7, 95% CI: 1.25, 6.12), parity (AOR = 6.7, 95% CI: 1.55, 12.6), history of abortion (AOR = 3.5, 95% CI: 1.63, 7.58), booking status (AOR = 5.8, 95% CI: 3.15, 9.72), and time of drug given (AOR = 4.9, 95% CI: 1.86, 13.22).

**Conclusion:**

It was discovered that severe preeclampsia and eclampsia had a high overall rate of unfavorable maternal outcomes. Promoting early antenatal care booking and formal education for women can reduce preeclampsia and eclampsia outcomes.

## Introduction

Preeclampsia is the new onset of proteinuria ≥0.30 g/24 h and hypertension ≥140/90 mmHg in a previously normotensive woman after 20 weeks of gestation (1, 2). Severe preeclampsia is a substantial clinical manifestation of preeclampsia that is marked by severity symptoms such as multiple organ failure, excretion of more than 5 gm of protein per 24 h, oliguria (<400 ml/hr), and a persistent rise in blood pressure (≥160/110 mmHg) (3). Eclampsia is a serious obstetric emergency with a new onset of grand mal seizure during pregnancy or postpartum in a woman having signs or symptoms of preeclampsia (4). Severe preeclampsia and eclampsia can lead to unfavorable maternal outcomes, including HELLP syndrome, hepatic subcapsular hematoma, acute kidney injury, liver rupture, placental abruption, and postpartum hemorrhage, potentially leading to maternal (5, 6). HELLP Syndrome is a syndrome, characterized by Hemolysis, Elevated Liver enzymes, and Low Platelet count, and is a severe form of preeclampsia (6, 7).

There are 295,000 maternal fatalities globally after pregnancy and childbirth, which is an unacceptably high rate (8). Roughly 86% of estimated global maternal deaths occurred in low-resource settings, such as Sub-Saharan Africa and Southern Asia, where the vast majority of these deaths (94%) took place (9). Approximately two-thirds (196,000) of maternal deaths occurred in Sub-Saharan Africa alone, with approximately one-fifth (58,000) occurring in Southern Asia (10). A global analysis of the cause of maternal mortality found that hypertensive disorders are associated with the second most common direct obstetric cause of death for mothers, accounting for 11%–14% of deaths worldwide, most of which occur in developing countries (11).

According to research done in the United States of America, pregnancy-induced hypertension complicates 2%–8% of pregnancies and is a major cause of maternal morbidity and mortality worldwide (12). Ethiopia is one of the fifteen nations that are on “a very high alert” for maternal death, according to the Fragile States Index (13). Despite tremendous efforts by the Ethiopian state government and national and international nongovernmental groups to lower it, maternal mortality remains a serious problem, with 267 deaths for every 100,000 live births (14).

According to the Ethiopian National Emergency Obstetric and Newborn Care review (EMONC), preeclampsia/eclampsia is the second most common cause of maternal morbidity and the third most common cause of maternal mortality (15). A study conducted at Ethiopia's Jimma University Hospital found that hemorrhage (54%) is the leading cause of maternal fatality, followed by pregnancy-induced hypertension (20%) (16).

Together with the World Health Organisation (10) and other nongovernmental organizations, the Ethiopian government has committed to lowering maternal mortality through several high-impact interventions at the facility and community levels. These interventions aim to remove barriers to safe motherhood services, such as unsafe traditional practices, inadequate care at health facilities, a lack of transportation options, and poor infrastructure (17). Despite significant efforts by several health sectors and funding bodies to decrease maternal mortality resulting from severe preeclampsia/eclampsia, the number of these deaths is continuously rising globally (18).

The aforementioned viewpoints from various nations indicate that the burden of severe preeclampsia/eclampsia is not restricted to maternal health issues, but also includes a noteworthy number of maternal deaths (18, 19). Research endeavors have attempted to examine the global trend, severity, and correlated variables of severe preeclampsia and eclampsia (20), but almost all these studies used secondary data and were not focused on investigating maternal outcomes.

Thus, the time of drug initiation was considered in this investigation, along with the features of the prior studies that were used to evaluate the outcomes of severe preeclampsia and eclampsia, along with associated factors and clinical aspects of maternal morbidity/mortality.

## Methods

### Study design and period

An institution-based cross-sectional study design was employed from April 1 to the last day of September 2018.

### Study setting and population

“The study's locations were the referral hospitals in the Amhara Regional State. The area is situated between latitudes 11^30′00″ N and longitude 38^30′00″ E in the northwest of Ethiopia.

17,221,976 people are living in the catchment area of this region, with 8,641,580 men and 8,580,396 women with 2,112,595 (12.27%) being urban residents (21, 22).

According to the regional state health bureau, there are 67 hospitals, 839 health centers, and 3,336 health posts. Five of these are referral hospitals: University of Gondar Teaching Referral Hospital, Felege Hiwot Referral Hospital, Dessie Referral Hospital, Debremarkos Referral Hospital, and Debrebirhan Referral Hospital. It is assumed that each referral hospital serves a catchment population of approximately 5 million people, has 200–400 beds, 2,000–4,000 deliveries annually, and 5–10 deliveries per day.

All severe preeclamptic and eclamptic mothers admitted in selected Referral Hospitals, in the Amhara region, North West Ethiopia, during the data collection period, were included in the study.

Women with other medical complications like anemia, epilepsy, renal disease, chronic hypertension, diabetic mellitus, cardiac disease, malaria, and pregnancy complications like twin pregnancy, and amniotic fluid disorders were excluded from the study.

### Sample size and sampling procedure

The research created a baseline for preeclampsia and eclampsia cases in the Amhara region by counting cases in the logbooks of randomly chosen referral hospitals in 2022. After adding a 10% non-response rate, 456 people made up the total sample size. Up until the necessary sample size was attained, all patients who were significantly preeclamptic or eclamptic were included (Table 1).

**Table 1:** Number of severe preeclamptic and eclamptic cases in selected referral hospitals of Amhara region, North West Ethiopia.

Referral hospitals	Severe preeclampsia/eclampsia cases/year	Average preeclampsia/eclampsia cases/month	preeclampsia/eclampsia cases/6 months
Flege hiwot referral hospital	348	29	174
Debre markos hospital	276	23	138
Dessie referral hospital	204	17	102
Total	828	69	**414**

Source: Logbook of randomly selected referral hospitals of Amhara region (2017).

### Data collection procedures and instrumentation

Face-to-face interviews with patients or family members of eclamptic moms, outcome observation, and a semi-structured questionnaire modified from the literature were used to gather data from patient charts. Language experts produced the questionnaire in English, had it translated into the Amharic form of the local language, and then had it translated back into English to ensure consistency. Two data collectors and one supervisor were in charge of gathering data for each hospital. The supervisors were MSc midwives from the relevant Referral Hospitals, while the data was gathered by BSC midwives from nearby health centers.

### Data quality control

To guarantee the quality of the data, the design of the data collection tool was given top priority. Before any data were collected, the validity and reliability of the questionnaire were reviewed by senior researchers from the University of Gondar. At the Debre Berhan Referral Hospital, a pre-test was given to twenty-three mothers, or five percent of the sample size, to assure clarity. The pre-test findings were used to determine what adjustments were needed. Training was provided to supervisors and data collectors regarding the project's objectives, collecting methods, and relevance. Throughout the data collection process, each data collector was monitored for any problems and provided with advice and any necessary corrections.

### Data processing and analysis

To ensure correctness, every piece of information was coded and double-checked. The data was then imported into Epi Info 7.2 and exported to SPSS version 26 for the study of data cleansing. To control the effect of confounders, a multivariable logistic regression model was utilized in conjunction with bivariable logistic regression to find out associations. To control confounders, variables with a *P*-value of less than 0.2 from the bivariable analysis were incorporated into the multivariable logistic regression model. In the multivariable logistic regression analysis, a variable was deemed statistically significant if its *P*-value was less than 0.05. Next, the odds ratio at a 95% confidence interval was calculated. The Hosmer and Lemeshow goodness of fit test was used to verify the binary logistic regression model's assumption.

### Ethical consideration

The University of Gondar School of Midwifery Ethical Review Committee approved the data collection application by the ethical approval number MIDW/10/489/2018, and permission from the university was obtained. After that, the Amhara Regional Health and Amhara Region Referral Hospitals received this letter of permission. Participants or their relatives provided written informed consent after being informed that participation would be voluntary and confidential.

## Results

### Socio-demographic characteristics

Of the 456 cases in this study with a 100% response rate, 46.7% had eclamptic conditions and 53.3% had severe preeclampsia. The age range of the participants was 17–45 years old, with a mean age of 28.3 years (SD ± 6.5 years). More than three-quarters of the participants, or 374 (76.1%), were Orthodox Christians. Greater than three-quarters of the participants, 349 (76.5%) were in the age group of 20–35 years. About 193 (42.3%) patients responded that they were unable to read/write. Most of the respondents, 429 (94.1%) were married of which nearly two third of them, 281 (61.6%) were housewives. More than half of the participants, 235 (51.5%) were urban dwellers. Before entering the hospital, 58.8% of patients underwent traditional treatments. 49.6% of participants said their family's monthly income was less than 2,037 Ethiopian Birr (Table 2).

**Table 2:** Socio-demographic characteristics among mothers with severe preeclampsia and eclampsia admitted, in amhara region referral hospitals, North West Ethiopia.

Variables	Frequency	Percent
Maternal age		
age ≤19	33	7.3
age 20–35	349	76.5
age ≥36	74	16.2
Religion		
Orthodox	347	76.1
Muslim	101	22.2
Protestant	7	1.5
Catholic	1	0.2
Marital status		
Married	429	94.1
Single	20	4.4
Divorced	4	0.9
Widowed	3	0.7
Educational status		
Unable to read and write	193	42.3
Able to read and write	38	8.3
Elementary school	56	12.3
Secondary school	71	15.6
College and above	98	21.5
Occupation		
Unemployed	16	3.5
Housewife	281	61.6
private worker	76	16.7
governmental worker	66	14.5
NGO	6	1.3
Student	11	2.4
Residence		
Urban	235	51.5
Rural	221	48.5
Traditional treatment use		
Yes	268	58.8
No	188	41.2
Monthly family income in ETB		
Income <2,037	226	49.6
Income 2,037–3,506	95	20.8
Income >2,037	135	29.6

NGO, non governmental organizations; ETB, ethiiopian birr.

### Obstetric characteristics

With a mean gestational age of 38 weeks, 303 (66.45%) participants were multigravidas, and 261 participants (57.2%) with multiparas were included in the study. A little over 49.4% of women were between 37- and 40 weeks gestation, 69.1% had had at least one ANC follow-up, and 75.9% had never had an abortion (Table 3).

**Table 3:** Obstetrical characteristics of severe preeclampsia and eclamptic patients among mothers admitted in amhara region referral hospitals, north west Ethiopia.

Obstetrics variables	Frequency	Percent
Parity		
Nully para	72	15.8
Premipara	123	27.0
Multipara	261	57.2
Gestational age in weeks		
GA 20–27	58	12.7
GA 28–36	86	18.8
GA 37–40	225	49.4
GA ≥41	87	19.1
ANC follow up		
No	141	30.9
Yes	315	69.1
Mode of delivery		
SVD	136	29.8
Instrumental	10	2.2
C/S	138	30.3
Vaginal delivery after induction augmentation	172	37.7
History of abortion		
Yes	346	75.9
No	110	24.1
Booking status		
Booked	164	36.0
Unbooked	292	64.0

ANC, antenatal care; SVD, spontaneous vaginal delivery; C/S, cesarean section.

### Time of occurrence

Nearly two-thirds, 288 (63.2%) cases of severe preeclampsia and eclampsia happened during the antepartum period, with 28.9% and 7.9% occurring during the intrapartum and postpartum periods, respectively.

### Maternal outcomes of severe preeclampsia and eclampsia

Of the 456 Amhara region referral hospital patients with severe preeclampsia and eclampsia, 37.7% had adverse maternal outcomes. About 56.9% had PPH, and 0.5% (which means 5 death/1,000 women) died as a result of problems related to eclampsia (Table 4).

**Table 4:** Maternal outcomes of severe preeclampsia and eclampsia among mothers admitted in Amhara Region Referral hospitals, North West Ethiopia.

Maternal outcomes	Favorable	284 (62.3%)	
	Unfavorable	172 (37.7%)	
Types of unfavorable outcomes (*n* = 172)		Frequency	Percent
Abruptio placenta		47	27.3
HELLP syndrome		68	39.5
DIC		31	18.0
Acute renal failure		95	55.2
PPH		98	56.9
Aspiration pneumonia		92	53.4
Pulmonary embolism		3	1.7
Pulmonary edema		9	5.2
Death		1	0.5

DIC, disseminated intravascular coagulation; PPH, postpartum hemorrhage.

### Factors associated with unfavorable maternal outcomes of severe preeclampsia and eclampsia

After adjusting for the effect of other variables in binary and multivariable logistic regression: educational status, residence, monthly family income, parity, history of abortion, booking status, and time of drug given were the significant factors of the outcome variable (Table 5).

**Table 5:** Bivariable and multivariable logistic regression results of factors associated with maternal outcomes of severe preeclampsia and eclampsia among women admitted in Amhara region Referral hospitals, North West Ethiopia.

Variables	Maternal outcomes		COR (95% CI)	AOR (95% CI)
	Unfavorable	Favorable		
Maternal age				
Age ≤19	27	6	3.6 (1.33, 9.80)*	3.9 (0.52, 29.47)
Age 20–35	104	245	0.3 (0.20, 0.57)**	1.5 (0.37,5.67)
Age ≥36	41	33	1	1
Educational status				
Unable to read &write	96	97	**6.5** (3.38, 12.37)**	**4.5** (1.95, 12.31)*
Read & write	16	21	**5.0** (2.07, 11.93)**	**4.3** **(1.57, 11.85)**
Elementary school	24	34	**4.6** (2.10, 10.10)**	**4.0** **(1.53, 10.76)**
Secondary school	23	47	**3.2** (1.48, 6.89)*	1.9 (0.72, 5.14)
College & above	13	85	1	1
Residence				
Rural	127	93	**5.8** (3.80, 8.82)**	**2.1** (1.17, 3.72)*
Urban	45	191	**1**	1
Monthly family income				
Income <2,037ETB	112	114	**7.3** (4.07, 13.09)**	**2.8** (1.25, 6.12)*
Income 2,037—3,506ETB	44	51	**6.4** (3.31, 12.40)**	**2.7** (1.14, 6.46)*
Income >3,507ETB	16	119	**1**	1
Gravidity				
Premigravida	76	77	2.1 (1.42, 3.17)**	1.9 (0.43, 8.30)
Multigravida	96	207	1	1
Parity				
Nully para	50	20	**6.5** (3.64, 11.73)**	**6.7** (1.55, 12.6)*
Premipara	49	73	1.8 (1.11–2.75)*	1.7 (0.72, 3.94)
Multipara	73	191	**1**	1
History of abortion				
Yes	70	41	**4.1** (3.06, 8.02)**	**3.5** (1.63, 7.58)*
No	102	243	**1**	1
Booking status				
Unbooked	146	146	**5.2** (3.23, 8.55)**	**5.8** (3.15, 9.72)*
Booked	26	136	1	1
When did the drug given				
Late (after they developed complications)	146	121	**7.6** (4.68, 12.21)**	**5.0** (1.86, 13.22)*
Early (before the onset of complications)	26	163	**1**	1

Bold values show the significant association between the dependent and independent variables.

1, Reference; ETB, ethiopian birr.

* *p*-value < 0.05.

** *p*-value < 0.001.

## Discussion

This cross-sectional study, conducted in an institution, aimed to evaluate the maternal outcomes of severe pre-eclampsia/eclampsia and related factors among women hospitalized at referral hospitals in the Amhara region of North West Ethiopia in 2018.

Among the 456 instances of severe preeclampsia and eclampsia, 172 (or 37.7%) resulted in poor maternal outcomes, with a 95% confidence interval (32.8%–42.3%) which means 62.3% of the study participants had no adverse maternal outcomes from preeclampsia and eclampsia. This study finding was in line with a study done in Addis Ababa (36%), Ethiopia (23). This commonality can result from comparable healthcare systems and socio-demographics but is lower than a study done in India (59%) (24) and in low- and middle-income countries (more than 50%) (25). The discrepancy could be the result of the time interval since improvements in healthcare infrastructure and policy changes occur over time. The other possible explanation might be also differences in access to quality healthcare, clinical experiences of healthcare providers, poverty, and cultural practices, leading to delays in seeking and receiving timely care during pregnancy and childbirth (26).

There was one maternal death (0.5%) which is lower than in studies conducted in Debre Berhan Referral Hospital (2.5%) (27), India (11.2%) (24), and Haiti (1.9%) (28). Similarly, a study showed that, 10%–15% of direct maternal deaths are associated with preeclampsia and eclampsia in low- and middle-income countries (29, 30). One possible explanation for this discrepancy could be the passage of time. As time passes, there is a chance that behaviors related to seeking medical attention, getting access to medications and facilities, and hiring qualified personnel will all improve and potentially reduce the number of deaths. The other possible explanation also could be the differences in health care systems such as triage, transport, and treatment facilities differences (31). In this study, sixty-eight (39.5%) respondents developed HELLPs syndrome which is lower than the study done in Addis Ababa town (63.96%) (32) and Tanzania (50.9%) (33) and a study done in low- and middle-income countries (67.1%) (25) but higher than the study done in Mettu Karl Referral Hospital, Ethiopia (12.4%) (34) and Iran (4.9%) (35). The presence of HELLP syndrome in the women who died of eclampsia was 90.6% in high-income countries compared with 47.6% in low-income countries (25). The accessibility, quality, and expertise of laboratory personnel in detecting liver enzymes, along with the reagents used, may account for the discrepancy. One of the variables linked to worse outcomes from severe pre-eclampsia/eclampsia is the maternal educational status.

Women who were unable to read/write were 4.5 times more likely to develop unfavorable maternal outcomes of severe pre-eclampsia/eclampsia when compared with women whose educational statuses were college and above (AOR = 4.5, 95% CI: 1.95, 12.31).

This finding is supported by other studies done in South East Nigeria (36) and Bangladesh (37). This may be explained by the fact that illiterate respondents had poor knowledge of health care and sought out traditional healers instead of seeking treatment from medical professionals; in contrast, respondents with college degrees and higher had access to more information about health and where to obtain care. This also indicates that the higher the women’s educational status, the lower the maternal unfavorable outcome of severe preeclampsia/eclampsia.

Women who live in rural areas were 2.1 times more likely to develop adverse severe preeclampsia and eclampsia maternal outcomes than their counterparts (AOR = 2.1, 95% CI: 1.17, 3.72).

This is in agreement with studies done in Jimma University Hospital, Ethiopia (38), South East Nigeria (36) and India (39). One possible explanation for this could be the disparity in health care-seeking behavior and access between urban and rural women; the former are likely to receive better care and hence have fewer difficulties.

Another variable that was positively associated with unfavorable maternal outcomes of severe preeclampsia/eclampsia was family monthly income. Those women whose monthly family income was <2,037 ETB were 2.8 times more likely to develop unfavorable maternal outcomes of severe preeclampsia/eclampsia compared with women whose monthly income was >3,507 ETB (AOR =  2.8, 95% CI: 1.25, 6.12). Respondents whose monthly family income is between 2,037–3,506ETB were 2.7 times more likely to develop unfavorable maternal outcomes of severe preeclampsia and eclampsia than those having monthly family income >3,507ETB (AOR = 2.7, 95% CI: 1.14, 6.46).

This finding is similar to other studies conducted in Bangladesh (37) and India (39). One explanation for this could be that, even if the service is free and patients can use ambulances to get to the referral hospitals, these services aren't always functional or available at night or on weekends. Those without sufficient funds may currently face challenges getting about because they are unable to pay for public transport. This results in service delays and unfavorable consequences.

Nulliparous mothers were 6.7 times more likely to develop unfavorable maternal outcomes of severe preeclampsia and eclampsia than their counterparts (AOR = 6.7, 95% CI: 1.55, 12.6).

This finding is in agreement with studies done in South East Nigeria, Sub-Saharan Africa (40), and Pakistan (36, 41).

The possible explanation could be the fact that nulliparous women are those not given alive newborns meaning they had placental hormones abnormality that could be a risk factor for severe preeclampsia and eclampsia, this in turn may lead to unfavorable outcomes (42) than multipara.

Those respondents having a history of abortion were 3.5 times more likely to develop unfavorable maternal outcomes of severe preeclampsia and eclampsia than their counterparts (AOR = 3.5, 95% CI: 1.63, 7.58).

This finding was supported by a study done in India (43). The possible explanation is that abortion may lead to abnormal placentation and release of placental factors that contribute to systemic endothelial dysfunction for the next pregnancy which might maximize the occurrence of unfavorable outcomes of severe preeclampsia and eclampsia. Those respondents who were unbooked in the referral Hospitals were 5.8 times more likely to develop unfavorable maternal outcomes of severe preeclampsia and eclampsia than the booked ones (AOR = 5.8, CI: 3.15, 9.72).

This finding is supported by the study conducted in South East Nigeria (36). This is possibly explained by the three delays (delay in health care seeking, delay in transportation, and delay in getting service) faced by unbooked mothers either at home, health posts, or health center and finally reach referral hospitals after they develop complications that their counterparts. Those mothers who were provided drugs lately were 5 times more likely to get unfavorable maternal outcomes of severe preeclampsia and eclampsia than their counterparts (AOR = 5.0, CI: 1.86, 13.22). This is possibly explained by the fact that unfavorable maternal outcomes of severe preeclampsia and eclampsia are preventable if diagnosed and treated early.

### Limitation and strength

The short follow-up period of the study design we utilized makes it impossible to anticipate the long-term effects of severe preeclampsia and eclampsia. There might be potential recall bias during data collection; difficulty in getting complete data from client records; and Lack of information on neonatal outcomes.

The study covers multiple referral hospitals, enhancing the generalizability of the findings within the region. This study could also be a basis for researchers for future interventional studies to identify the neonatal and maternal outcomes of preeclampsia and eclampsia.

## Conclusion and recommendation

The study reveals high rates of adverse maternal outcomes in Amhara regional state referral hospitals due to severe preeclampsia and eclampsia, with factors like educational status, income, abortion history, booking status, residence, and drug administration time.

Empowering women through formal education, and promoting early antenatal care booking is recommended to decrease the poor outcome of preeclampsia and eclampsia. Again, further interventional research is recommended to identify the neonatal and maternal outcomes of preeclampsia and eclampsia.

## Data Availability

The original contributions presented in the study are included in the article/Supplementary Material, further inquiries can be directed to the corresponding author.
